# Bacterial contamination and infection control practices in the mortuary at Mbarara Regional Referral Hospital, Uganda

**DOI:** 10.1017/ash.2025.10131

**Published:** 2025-09-23

**Authors:** Edwina Patience Wagido, Adima Tom, Niwamanya Prosper, Tushabe Alfred, Tusiime Martha Genevieve, Okeny Christopher Wagido, Akuu Susan Langoya, Ssenkumba Brian, Kiconco Ritah, Pauline Petra Nalumaga, Mitala Yekosani, Atwine Raymond, Charles Bagenda, Rugera Simon Peter, Birungi Abraham

**Affiliations:** 1 Department of Medical Laboratory Science, Mbarara University of Science and Technology, Mbarara City, Uganda; 2 Department of Pathology, Mbarara Regional Referral Hospital, Ministry of Health, Mbarara City, Uganda; 3 Department of Microbiology, Mbarara University of Science and Technology, Mbarara City, Uganda; 4 Department of Pathology, Busitema University, Faculty of Health Sciences, Mbale City, Uganda

## Abstract

**Background:**

Increasing evidence demonstrates that medical personnel and the clinical environment such as surfaces and equipment are often sources of infections. However, limited data exists on the bacterial contamination of the hospital mortuary environment within a hospital setting.

**Objective:**

This study aimed to determine bacterial profiles, assess antimicrobial susceptibility patterns, and evaluate infection prevention and control (IPC) practices at the Mbarara Regional Referral Hospital (MRRH) mortuary in southwestern Uganda.

**Methods:**

This was a cross-sectional study involving qualitative and quantitative data collection methods. Ninety-three (93) surface swab samples from the inanimate surfaces were collected. Using standard operating protocols, the collected samples were processed at the microbiology laboratory. All isolates were cultured and identified by Gram staining and biochemical tests. Antimicrobial susceptibility testing was done on each isolate following the Kirby-Bauer disk diffusion method. Data was entered and analyzed using SPSS version 26 and results were explained by percentages and tables.

**Results:**

*Klebsiella species* were the most predominant isolated bacteria (44.7%) followed by *Staphylococcus aureus* (40.3%). Methicillin-resistant *Staphylococcus aureus* (MRSA) was detected at a high frequency (98%). Resistance patterns showed that most isolates exhibited multidrug resistance, with high resistance to commonly used antibiotics. However, Azithromycin and Ciprofloxacin demonstrated the highest efficacy against both Gram-positive and Gram-negative isolates.

**Conclusion:**

Inanimate surfaces and equipment of the mortuary are heavily contaminated with multidrug-resistant bacteria such as Methicillin-resistant *Staphylococcus aureus*. Improper disinfection and waste segregation may contribute to bacterial contamination in the mortuary. Regular and enhanced cleaning with disinfectants, proper use of clean aprons, and displaying safety signage for workers and visitors can help improve infection prevention and control.

## Introduction

Occupational and environmental diseases contribute to an estimated 2 million deaths annually, with approximately 6,400 daily fatalities globally.^1,2^ Healthcare environments, including hospitals and mortuaries, serve as significant reservoirs for clinically important bacterial pathogens, including multidrug-resistant (MDR) organisms.^3^ These bacteria pose a severe threat to healthcare workers, visitors, and the broader community due to their potential for cross-transmission and persistence on inanimate surfaces and medical devices.^4^

Hospital-acquired infections (HAIs) remain a major public health concern, with bacterial cross-contamination playing a crucial role in their spread. While direct patient-to-patient transmission has been well-documented, increasing evidence suggests that healthcare personnel and contaminated environmental surfaces such as medical devices and door knobs significantly contribute to infection propagation.^5^ The emergence and spread of MDR pathogens, such as methicillin-resistant Staphylococcus aureus (MRSA) and carbapenem-resistant *Klebsiella pneumoniae*, further complicate infection control efforts.^6^ The increase of multi-drug resistance (MDR) strains in hospital settings, particularly in developing nations including Uganda, is a growing concern that hinders healthcare-associated infection (HCAI) management.^7^ The inappropriate use of antibiotics accelerates the selection of resistant strains, which thrive in healthcare settings with suboptimal hygiene and infection control measures. Mortuary environments, in particular, provide a unique setting where microbial contamination from deceased bodies, healthcare workers, and inadequate sanitation practices can facilitate the persistence of pathogenic bacteria.^8^

Infection prevention and control (IPC) practices play a crucial role in reducing microbial contamination and controlling the spread of resistant pathogens.^9^ Strict adherence to standard operating procedures (SOPs) and proper waste management are essential strategies for mitigating infection risks in high-contamination areas such as mortuaries.^10^ However, limited data exist on the prevalence of bacterial contamination and the effectiveness of IPC practices within hospital mortuaries in Uganda.

This study aims to bridge the knowledge gap by determining the bacterial profiles present in the mortuary environment of Mbarara Regional Referral Hospital (MRRH) and assessing antimicrobial resistance patterns. Additionally, it evaluates current IPC practices and highlights areas for improvement to enhance biosafety and protect both healthcare workers and visitors.

## Methods

### Study site and population

This was a cross-sectional study involving qualitative and quantitative methods of data collection conducted in MRRH mortuary, located in Mbarara City, southwestern Uganda at approximately 267 km from Kampala City along Kampala—Kasese Highway on the latitude 0^o^37’3” S and longitude 30^o^39’25” E. The Mortuary serves Southwestern Uganda with catchment areas of Mbarara district, Kiruhura, Isingiro, Kazo, Ibanda, and Rwampara working on an average of 1825 dead bodies annually, with an average of 18,250 visitors per year.

### Data collection tools

A pretested structured questionnaire consisting of closed and open-ended questions were developed and focused on identifying the practices done to curb the growth of infectious pathogens in the mortuary. An observation checklist was developed focused on identifying gaps in order to give recommendations in reference to health and safety practices.^10^

### Sample collection

A total number of ninety-three (93) swabs were collected from the mortuary room, autopsy room, and mortuary office from inanimate surfaces and equipment in all rooms. The surfaces included sinks, beds, doorknobs, switches, mortuary office tables, freezer handles, autopsy tables, floor, report writing stations, workbench tops, taps, walls, doors, cabinets, autopsy tables, and drainage tubes. The equipment includes tools, thermometer, carrier, trolley, plastic body lift, blades, body tray, weighing scale, embalming can, disposable containers, jerry cans, chemical storage bottles, carrier, and blades. Surfaces such as fridge compartments, corpses, and visitors were excluded considering they were the mostly accessed surfaces and equipment. Samples were collected using swab sticks moistened with normal saline. The areas were swabbed in two directions at right angles to each other in a close zigzag pattern at each site, rotating the swabs during sampling to ensure that the full surface of the swab is utilized. After swabbing, the sticks were placed in peptone water and transported to the laboratory for processing.

### Processing and identification of isolates

Samples were processed from the microbiology laboratory of Mbarara Regional Referral Hospital. The peptone water containing the samples was incubated for 24 h at 37 °C for growth. Growth was observed as turbidity while the absence of turbidity was considered as no growth.^11^ Samples from the peptone water were inoculated using a sterile wire loop onto 5% blood agar and MacConkey agar (BioLab Inc), and the inoculated plates were incubated at 37 °C for 24 h. Gram staining and standard biochemical tests identified the characteristics of isolated bacteria that showed significant growth. Gram-positive cocci were identified by Gram staining and catalase, coagulase tests, Mannitol salt agar fermentation, and DNASE tests. Gram-negative bacterial isolates were identified using a series of biochemical tests including indole, citrate, motility, urease, oxidase, and triple sugar iron.^12,13^

### Antimicrobial susceptibility

Antibacterial susceptibility tests were performed using the Kirby-Bauer disk diffusion method on the Mueller-Hinton agar (Biolab Inc) and interpreted according to the Clinical Standard

Institute (CLSI) guidelines 2020.^14^ Eight antibiotic discs (Biolab Zrt) were used, which included Ciprofloxacin (5 µg), Tetracycline (30 µg), Azithromycin (15 µg), Piperacillin-Tazobactam (100/10 µg) Chloramphenicol (30 µg), and Penicillin (10 µg) were used for both the Gram-positive and Gram-negative bacteria. Erythromycin (15 µg) and Cefoxtin (30 µg) were used on only *Staphylococcus aureus.*

### Quality control

Aseptic techniques in all steps of sample collection and inoculation onto culture media were ensured to minimize contamination. *Escherichia coli* (ATCC 25922) and *Staphylococcus aureus* (ATCC 25923) were used as control strains. A pretested checklist and questionnaire were used.

### Ethical considerations

Ethical approval was obtained from Mbarara University of science and Technology Research Ethics Committee under number MUST-2023-884 before conducting the study. Administrative approval from the director of MRRH and the medical superintendent of the MRRH Mortuary were sought.

### Data analysis

The data collected was entered in an Excel spreadsheet (Microsoft Office Professional Plus 2013, version 15.0.4675.1003, Microsoft Inc. USA) and then imported into Statistical Package for the Social Sciences (IBM SPSS Inc. version 23) software. Descriptive statistics were used to describe the bacterial growth patterns, antibiogram, and infection and prevention practices using charts, tables, frequencies, means ± standard deviations (SDs) for continuous variables as well as frequencies and proportions to describe categorical variables.

## Results

### Bacteria profile

**Figure 1.**
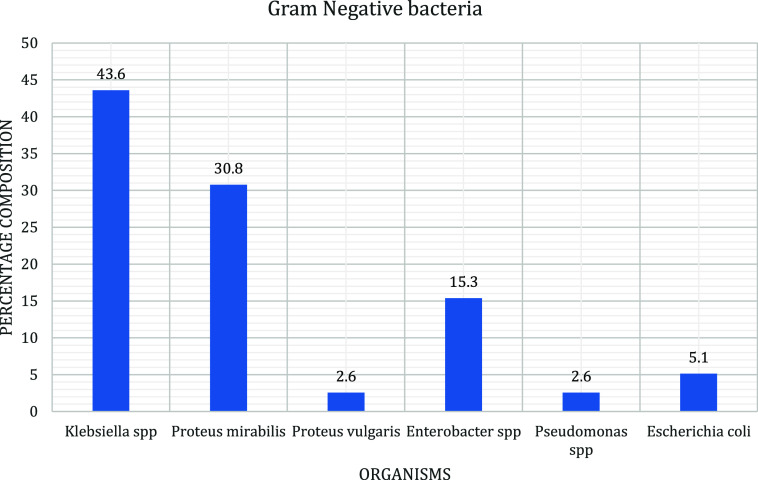
A bar graph showing Gram-negative bacterial isolates from inanimate surfaces of the mortuary at Mbarara Regional Referral Hospital.

After culture, 53 (57%) of the 93 samples from the inanimate surfaces showed significant bacterial growth with diversity of microbial pathogens. Among the samples with bacterial growth, 13 samples had mixed growth while 40 samples had pure growth; therefore, 66 bacterial isolates were obtained. Majority of the isolates were Gram-negative bacteria with 39 (60%) of overall pathogens while 27 (40%) were Gram-positive bacteria particularly *Staphylococcus aureus*. Of the Gram- negative isolates, *Klebsiella pneumoniae* (43.6%) and *Proteus mirabilis* (30.8%) had the highest frequencies of occurrence whereas *Proteus vulgaris* and *Pseudomonas aeruginosa* had the least frequencies of occurrence at 2.6% (Figure 1)

### Antibiogram

The only isolated Gram-positive organism in this study, *Staphylococcus aureus* displayed resistance to the most commonly used antibiotics namely Tetracycline, Tazobactam+Piperacillin, Chloramphenicol, Erythromycin, and Cefoxitin (Table 1). The Gram-negative bacteria were relatively sensitive to most antibiotics expect *Pseudomonas aeruginosa* (Table 2).

**Table 1.** Showing resistance profiles of *Staphylococcus aureus* isolated from inanimate surfaces of the mortuary at Mbarara Regional Referral Hospital

*S.aureus* = 27	CIP		AZM		TE		TZP		C		P		E		FOX	
	S	R	S	R	S	R	S	R	S	R	S	R	S	R	S	R
	23(85%)	4(15%)	25(92%)	2(8%)	19(70%)	8(30%)	2(8%)	25(92%)	2(8%)	25(92%)	4(15%)	23(85%)	23(85%)	4(15%)	1(4%)	26(96%)

**KEY**: CIP-Ciprofloxacin, AZM-Azithromycin, TE-Tetracycline, C-Chloramphenicol, P-Penicillin, E-Erythromycin, FOX-Cefoxtin.

**Table 2.** Resistance profiles of Gram-negative bacteria isolated from inanimate surfaces of the mortuary at Mbarara Regional Referral Hospital

	CIP		AZM		TE		TZP		C	
	S	R	S	R	S	R	S	R	S	R
***Klebsiella* spp**	8(47.1%)	9(52.9%)	16(94.1%)	1(5.9%)	9(52.9%)	8(47.1%)	4(23.5%)	13(76.5%)	11(64.7%)	6(35.3%)
***Proteus Vulgaris***	0(0%)	1(100%)	1(100%)	0(0%)	1(100%)	0(0%)	0(0%)	1(100%)	0(0%)	1(100%)
***Enterobacter* spp**	5(83.3%)	1(16.7%)	4(66.7%)	2(33.3%)	4(66.7%)	2(33.3%)	1(16.7%)	5(83.3%)	5(83.3%)	1(16.7%)
***Proteus mirabilis***	54.5	45.5	81.8	18.2	45.5	54.5	9.1	90.9	54.5	45.5
***Escherichia coli***	2(100%)	0(0%)	1(50%)	1(50 %)	2(100%)	0(0%)	0(0%)	2(100%)	0(0%)	2(100%)
***Pseudomonas* spp**	0(0%)	1(100%)	0(0%)	1(100%)	0(0%)	1(100%)	0(0%)	1(100%)	0(0%)	1(100%)

**KEY**: CIP-Ciprofloxacin, AZM-Azithromycin, TE-Tetracycline, C-Chloramphenicol, P-Penicillin.

### Infection, prevention and control practices

#### Sanitary inspection of the mortuary environment

A standard checklist to inspect the sanitary environments for potential attributes that could result in the spread of microbial pathogens on inanimate surfaces. According to our findings, there was good engineering control with the provision of functional fume hood for sample grossing with running water for protection from aerosols. There were sufficient ventilations and lightings of the facility. There were available hand sanitizers and handwashing facilities for the occupants and visitors of the facility. However, the notable non-conformities that predisposed this facility to microbial contamination included the irregularity in cleaning the mortuary surface, inadequate supply of waste bins and consequently poor waste segregation, and lack of well-displayed visual signage to guide visitors and relatives of the deceased who come to this facility. The use of clean aprons as an infection prevention and control tool showed statistical significance (.036) while other infection prevention and control practices did not. The findings are as summarized in the frequency distribution table 3 below.

**Table 3.** Frequency distribution table showing the infection prevention and control measures employed at the mortuary at Mbarara Regional Referral Hospital, 2024 as reported by the respondents

		Frequency	Percentages	P values
**Occupation**	Admin clerk Porter Lab. Technician Pathologist	1/8 1/8 1/8 5/8	12.5 12.5 12.5 62.5	.659
**Work experience**	<2 years 2–5 years 6–10 years >10 years	1/8 3/8 3/8 1/8	12.5 37.5 37.5 12.5	.217
**Occurrence of Cut wounds**	Slightly common Very common	3/8 5/8	37.5 62.5	.206
**Occurrence of puncture wounds**	No Slightly common Very common	1/8 3/8 4/8	12.5 37.5 50.0	.264
**Following SOPs**	Yes No Not sure	5/8 2/8 1/8	62.5 25.0 12.5	.587
**Access to occupational health and safety Act**	Yes No Not sure	1/8 5/8 2/8	12.5 62.5 25.0	.449
**Knowledge of system of accidents reporting**	Yes No	4/8 4/8	50.0 50.0	.102
**Use of Personal protective equipment (PPE)**	Yes No	5/8 3/8	62.5 37.5	.673
**Use of face mask**	Yes No	7/8 1/8	87.5 12.5	.064
**Cleaning of PPEs**	Regular Irregular	2/8 6/8	25.0 75.0	.346
**Cleaning plastic apron**	**Yes** **No**	**3/8** **5/8**	**37.5** **62.5**	**.036**
**Use of disinfectants**	Yes No	6/8 2/8	75.0 25.0	.673
**Safety induction**	Yes No	7/8 1/8	87.5 12.5	.064

## Discussion

### Bacteria profile

This study highlights the significant bacterial contamination on inanimate surfaces and equipment within the mortuary at Mbarara Regional Referral Hospital, with a predominance of g bacteria, particularly *Klebsiella pneumoniae*. The presence of multidrug-resistant organisms, including MRSA, raises urgent concerns about infection control and antibiotic resistance in healthcare environments. These findings align with previous research demonstrating that hospital settings (15, 16), especially mortuaries that serve as reservoirs for persistent and resistant bacterial strains due to inadequate sanitation and improper antibiotic use.^17^

The results from the study showed that 53 (57%) of 93 of the inanimate surfaces and equipment of the mortuary were contaminated. The observed percentages of bacterial contamination could be explained by insufficient cleaning protocols due to non-conformity as seen in the observational checklist that predisposed this facility to microbial contamination. These included the inadequate supply of waste bins and consequently poor waste segregation.

Bitew et al,^16^ while studying bacterial isolates in the mortuary in Ethiopia reported more g (51.1%) isolates than g bacteria.^15^ Furthermore, another study also conducted in Ethiopia revealed *E.coli* as the most common isolate from inanimate surfaces.^18^ The similarity in isolated organisms maybe due to similar hospital-environment conditions in East Africa. This current data from this study indicates how the hospital environment increases the risk of nosocomial transmission of HAIs.

### Antibiogram

*Pseudomonas aeruginosa*, a known opportunistic pathogen identified in this study showed absolute resistance to all tested antibiotics, underscoring the challenge of managing infections caused by such strains. Pseudomonas species possess intrinsic resistance mechanisms and a remarkable ability to acquire additional resistance traits,^19^ making them difficult to eradicate from hospital environments. The observed resistance in this study might be linked to the widespread indiscriminate use of these antibiotics in the study area as they are cheap and easily available with limited policies. The release of these bacteria into the environment can exacerbate the problem of antibiotic resistance, posing a risk to public health.^20^

*Staphylococcus aureus* expresses many virulence factors and often cause skin and soft tissue infections, pneumonia, heart valve infections, bone infections soft tissue infections, and bloodstream infections.^21^ MRSA is well-documented for its ability to persist on surfaces and medical devices, contributing to healthcare-associated infections (HAIs). The 98% resistance rate to methicillin observed in this study is alarmingly high, suggesting gaps in antibiotic stewardship and hygiene compliance. This finding aligns with previous research identifying MRSA as a persistent contaminant in healthcare settings, often linked to inadequate sanitation and improper handling of personal protective equipment.^22–27^

Most isolates were resistant to one or more antibiotics tested in the present study. This may also be due to the misuse, overuse, and inappropriate antibacterial agents. Moreover, the mortuary receives corpses from many districts and distant rural villages. These corpses could have taken different antibiotic treatments from the general practitioners and nurses or from over-the-counter, usually with inappropriate doses, before coming to the hospital and succumbing to death. Therefore, the finding sounds the alarm for the implementation of a nationwide antimicrobial surveillance and in vitro susceptibility testing at all levels of private and government hospitals with strict adherence to antibiotic policy to inhibit the spread of drug-resistant microbes in the country.^25^

### Infection, prevention and control

The Ministry of Health in Uganda emphasizes that IPC is a vital component of the healthcare system, as it encompasses standard protocols designed to reduce and prevent the spread of infectious diseases among patients, healthcare workers, and visitors in medical facilities provided these protocols are properly followed.^28^

IPC practices are essential in all settings to reduce the risk of acquiring infections and to prevent the transmission of diseases to others.^29^ The study also assessed IPC practices and identified several deficiencies, including irregular cleaning schedules, inadequate waste segregation, and inconsistent PPE usage. Cleaning and decontamination practices were found to be suboptimal, with some staff members using disinfectants at unknown concentrations. These lapses create an environment conducive to bacterial survival and transmission.

There were statistically significant associations (*P* = .036) between cleaning of plastic aprons by mortuary staffs and surface contamination. This demonstrates that cleaning the aprons significantly decreases the level of surface contamination because contaminated plastic aprons act as fomites in the dissemination of pathogens on work surfaces. In a similar study done in Kenya, the use and cleaning of gowns and coats was also statistically significant with their p-values .02 and .01 respectively.^30^ The study established that the availability, use, and cleaning of PPE was important in reducing the bacterial loads in the hospital environment. However, another study done in South Africa, cleaning of aprons was statistically insignificant which was contrary to our study.^2^ The use of unknown/unstandardized concentration of sodium hypochlorite solution cannot be neglected although this observation was not statistically significant (*P* = .673). It is imperative that immediate attention is given to addressing these gaps in IPC practices, including ensuring proper disinfectant concentrations, regular cleaning and maintenance of PPE. Additionally, continuous training and monitoring of mortuary staff on best IPC practices will help mitigate the risks associated with pathogen transmission.

## Conclusion

This study emphasizes the critical need for improved IPC measures in hospital mortuary settings to prevent the high occurrence of multidrug-resistant bacterial contamination. Improved hygiene techniques, appropriate antibiotic usage, and adequate staff training will be significant in reducing the prevalence of HAIs to address these issues in order to create a safer mortuary environment.

## Recommendation

Regular cleaning of the mortuary with detergents and subsequently with disinfectants such as 5% sodium hypochlorite solution which has antimicrobial activity, removes fixed and dried microorganisms, leaves no residues, and is effective.

Proper waste segregation and management through the use of appropriately color-coded waste containers and regularly emptying them.

Continuous training all the mortuary staff in IPC practices and guidelines and putting in place a system of monitoring whether IPC practices are being monitored.

The facility should provide all the relevant safety signage and posters placed at strategic locations for both workers and visitors.

Further studies should be done on investigating the prevalence and incident of occupational diseases and injuries, the impact of temperature on the health of employees, the quality of air or efficiency of the ventilation system in the mortuaries, the efficiency of the PPE used by the mortuary workers, and other microbial population such as parasites, virus, molds, and yeasts.

## Data Availability

The analyzed datasets are available from the corresponding author upon request.
